# Zoonotic disease research in East Africa

**DOI:** 10.1186/s12879-018-3443-8

**Published:** 2018-11-03

**Authors:** Naomi Kemunto, Eddy Mogoa, Eric Osoro, Austin Bitek, M. Kariuki Njenga, S. M. Thumbi

**Affiliations:** 1 Paul G. Allen School for Global Animal Health, Washington State University, Pullman, USA; 2 Washington State University Global Health Program Kenya, Nairobi, Kenya; 3 Faculty of Veterinary Medicine, University of Nairobi, Nairobi, Kenya; 4 Food and Agriculture Organization of the United Nations, Nairobi, Kenya; 5 Center for Global Health Research, Kenya Medical Research Institute, Nairobi, Kenya

**Keywords:** Zoonoses, East Africa, Endemic, Epidemic, Research

## Abstract

**Background:**

The East African region is endemic with multiple zoonotic diseases and is one of the hotspots for emerging infectious zoonotic diseases with reported multiple outbreaks of epidemic diseases such as Ebola, Marburg and Rift Valley Fever. Here we present a systematic assessment of published research on zoonotic diseases in the region and thesis research in Kenya to understand the regional research focus and trends in publications, and estimate proportion of theses research transitioning to peer-reviewed journal publications.

**Methods:**

We searched PubMed, Google Scholar and African Journals Online databases for publications on 36 zoonotic diseases identified to have occurred in the East Africa countries of Burundi, Ethiopia, Kenya, Tanzania, Rwanda and Uganda, for the period between 1920 and 2017. We searched libraries and queried online repositories for masters and PhD theses on these diseases produced between 1970 and 2016 in five universities and two research institutions in Kenya.

**Results:**

We identified 771 journal articles on 22, and 168 theses on 21 of the 36 zoonotic diseases investigated. Research on zoonotic diseases increased exponentially with the last 10 years of our study period contributing more than half of all publications 460 (60%) and theses 102 (61%) retrieved. Endemic diseases were the most studied accounting for 656 (85%) and 150 (89%) of the publication and theses studies respectively, with publications on epidemic diseases associated with outbreaks reported in the region or elsewhere. Epidemiological studies were the most common study types but limited to cross-sectional studies while socio-economics were the least studied. Only 11% of the theses research transitioned to peer-review publications, taking an average of 2.5 years from theses production to manuscript publication.

**Conclusion:**

Our findings demonstrate increased attention to zoonotic diseases in East Africa but reveal the need to expand the scope, focus and quality of studies to adequately address the public health, social and economic threats posed by zoonoses.

## Background

Nearly two-thirds of human infectious diseases and majority of emerging infectious diseases exerting heavy public health and economic burden to the global community originate from animals [1–3]. Based on their impact and epidemiological characteristics, these zoonotic diseases have been categorized into the more common endemic zoonoses such as salmonellosis, brucellosis and leptospirosis which are responsible for more than 2.2 million human deaths and 2.4 billion cases of illness annually, and the less common epidemic and emerging zoonoses such as anthrax, Rift Valley fever, Ebola, Zika which either occur in sporadic outbreaks in neglected populations or that are new or re-appearing with increased incidence or geographical range [4].

Zoonoses and diseases recently emerged from animals have been estimated to contribute more than a quarter of the disability-adjusted life years (DALYs) lost to infectious diseases in low income settings such as sub-Saharan Africa, and less than 1% in high income countries [5]. The attention given to zoonotic diseases has however focused more on emerging zoonoses that pose global economic and health threats and less on the endemic zoonotic diseases which tend to occur among populations with little political voice [6–8].

The emergence of zoonotic diseases has been accompanied by research to understand when, how, and where they emerge, their pathogenesis and progression, diagnostics and treatment, and strategies for their prevention and control [9, 10]. Taking the example of the recent emergence of HIV, research has played a critical role to understand when and from where HIV emerged, understanding its transmission and pathogenesis, development of anti-retroviral drugs, and prevention modalities that have made its control as a global pandemic possible [10]. Thorough research, environmental, biological, economic and social drivers of disease emergence have been identified [11, 12], hotspots for emergence of wildlife and vector-borne zoonotic diseases identified to be regions in the lower latitudes [3].

Here we focus on East Africa region which has been identified as one of the zoonoses hotspot regions with a high prevalence of endemic zoonotic diseases [4], and where like in the rest of sub-Saharan Africa has a large rural population that lives in close proximity with livestock and wildlife. In order to understand the regional research trends on zoonotic diseases, we conduct a systematic assessment of published literature on zoonotic diseases in the region and theses research in Kenya, characterize the publications, and determine the transition of theses research to peer-reviewed publications.

## Methods

### Selection of zoonotic diseases and search strategy

We used a list of 36 zoonotic diseases suspected or known to be present in the East Africa region identified by a team of public health and veterinary experts in zoonoses in Kenya (see Table 3 in the referenced article) [13]. Using PubMed, Google scholar and African Journal of Science, we searched for published articles on these zoonotic diseases in Kenya, Uganda, Tanzania, Burundi, Rwanda and Ethiopia for the period between 1920 and 2017. The search terms included a combination of the zoonotic disease and the East Africa region and then in the specific country e.g. ‘Anthrax East Africa’; ‘Anthrax Kenya’; ‘Anthrax Tanzania’; ‘Anthrax Uganda’; ‘Anthrax Ethiopia’; ‘Anthrax Rwanda’; ‘Anthrax Burundi’. References in the identified articles were reviewed for additional publications. Only articles in English or in French with an English abstract on research on any of the 36 zoonotic diseases conducted in any of the East Africa countries were considered for further evaluation.

In addition, we conducted a review of MSc and PhD theses submitted to five major universities in Kenya (University of Nairobi, Jomo Kenyatta University of Agriculture and Technology, Moi University, Egerton University and Kenyatta University) that have offered graduate training in either medical, veterinary, or public health for at least 20 years. We included theses research available in two biomedical research institutions in Kenya: the Kenya Medical Research Institute and Institute of Primate Research.

The theses search at the Universities and research institutions was carried out in two stages. The first stage included a systematic search of online repositories of the various study institutions queried using search terms for each of the specific zoonotic diseases of interest e.g. ‘Anthrax’, ‘Trypanosomiasis’. Stage two entailed visiting each institution’s library and conducting a physical verification of the theses and dissertations, and updating the list generated in stage-one. Information including thesis title, author names and year of degree award were counter-checked and verified. Researchers at the Kenya Medical Research Institute and the Institute of Primate Research were contacted and requested to provide information on relevant theses and dissertations awarded in any of the five study universities available in their institutions’ libraries. The theses and dissertations selected were based on the following inclusion criteria: i) theses and dissertations with data on any of the 36 zoonotic diseases of interest ii) research carried out in Kenya between the period 1970–2016. For diseases such as trypanosomiasis, schistosomiasis and leishmaniasis with both zoonotic and non-zoonotic species, only studies specific to zoonotic species were considered.

### Data management and cleaning

Data variables extracted from the identified articles and theses included author, title, country, disease studied, pathogen species, journal, affiliation institution of first author, year of publication/year degree award, University name, subject of the study and study species (human or livestock or wildlife). By comparing details of the theses and journal publications retrieved, we determined theses research that had been published in peer-reviewed journals.

For analysis, the studies were classified into any of three categories: laboratory, epidemiology or socio-economic studies. A study was considered a laboratory study if it was an experimental study conducted within a laboratory setting, or studies developing, testing or comparing diagnostic methods. Studies were considered epidemiological if they determined the distribution, prevalence, incidence, mortality and morbidity rate, associated risk factors, knowledge and practices and control and prevention of the disease. Epidemiological studies were further classified based on the epidemiological study design to determine the most common design methods used. Studies determining the socio-economic impact of zoonotic diseases in either humans, animals or both were classified under socio-economic category. All the data were entered into an Excel spreadsheet, and imported in to R statistical software for analysis [14].

## Results

### Study selection

**Fig. 1:**
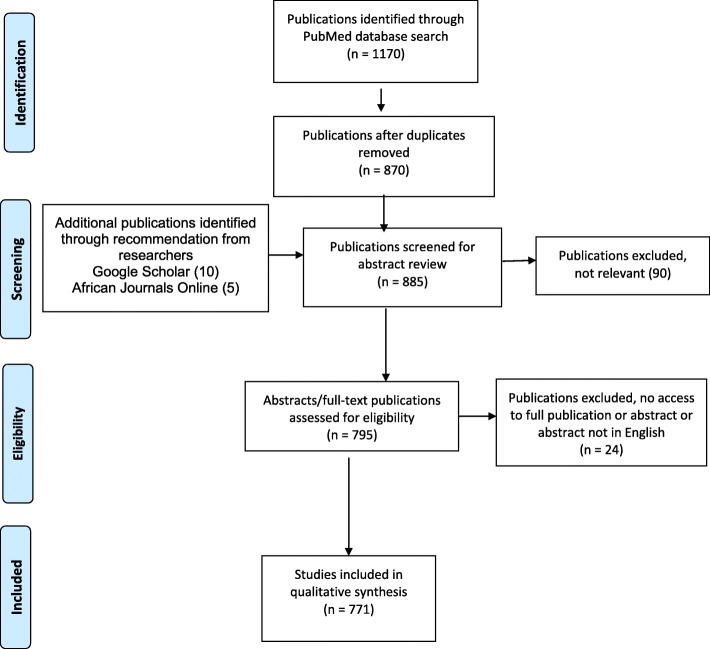
Flow diagram summarizing the selection process of publications included in the review

A total of 1170 articles were retrieved from PubMed and an additional 10 from Google Scholar and five from African Journals Online databases. Out of the 1185 articles, 390 were removed for being either duplicates, research conducted outside of East Africa or on diseases not selected for this study. A further 24 articles were dropped since full publications or abstracts were unavailable or not in English, remaining with 771 publications for analysis, Fig. 1. The 771 journal articles covered 22 of the 36 zoonotic diseases evaluated, Table 1.

**Table 1:** Summary of the zoonotic diseases from publications in East Africa and theses from Kenyan Universities analysed in this study

	Disease	Publications		Theses	
		Frequency	%	Frequency	%
1	Trypanosomiasis	166	21.5	14	8.3
2	Brucellosis	87	11.3	29	17.3
3	Rift Valley Fever	85	11	17	10.1
4	Rabies	62	8	4	2.4
5	Echinococcosis	56	7.3	21	12.5
6	Cysticercosis	48	6.2	11	6.5
7	Cryptococcosis	44	5.7	1	0.6
8	Campylobacteriosis	40	5.2	5	3
9	Bovine tuberculosis	38	4.9	9	5.4
10	Dengue	31	4	4	2.4
11	Ebola	29	3.8		
12	Leptospirosis	25	3.2	4	2.4
13	Q fever	19	2.5		
14	Anthrax	13	1.7	1	0.6
15	Marburg	9	1.2		
16	Schistosomiasis	5	0.6	1	0.6
17	Mers-Cov	4	0.5		
18	Cyclosporiasis	3	0.4		
19	Crimean-Congo hemorrhagic fever	3	0.4	1	0.6
20	Aspergillosis	2	0.3	6	3.6
21	West Nile	1	0.1		
22	Leishmaniasis	1	0.1	1	0.6
23	Salmonellosis			17	10.1
24	Influenza virus			9	5.4
25	Cryptosporidiosis			9	5.4
26	Toxoplasmosis			3	1.8
27	Rickettsia			1	0.6

Our search terms identified 326 theses and dissertations from the online digital repositories of the five study universities in Kenya. Physical visits retrieved an additional 12 theses from the university libraries, 36 from the Kenya Medical Research Institute, and 15 from the Institute of Primate Research in Kenya. From the total of 389 theses and dissertations identified, 221 were dropped for either being duplicates or not meeting the inclusion criteria. In total 168 theses covering 21 of the 36 zoonotic diseases evaluated were included in the analysis, Table 1.

### Published data research trends

**Fig. 2:**
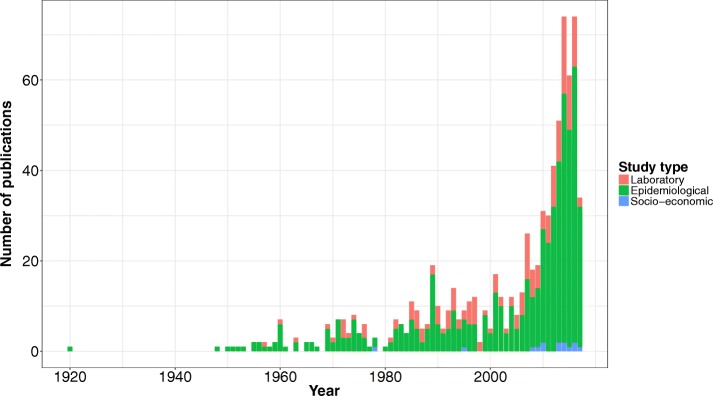
Diagram showing the annual publications on zoonotic diseases in East Africa region by study type

The number of publications on zoonotic diseases from the region has been increasing by year, with 460 (60%) published in the last 10 years. Epidemiological studies were the most common studies accounting for 585 (76%) of all publications reviewed, while laboratory studies were 172 (22%) and socio-economic studies 14 (2%), Fig. 2. By analyzing a random sample of 20% of the studies categorized as epidemiological, we found 75% of the study designs used were cross-sectional or case reports, 15% longitudinal study designs and the remaining case-control studies, reviews and modelling papers.

**Fig. 3:**
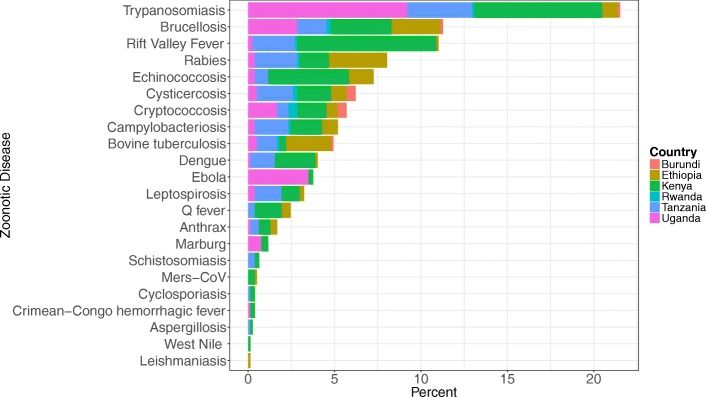
Diagram showing proportion of publications on the 22 zoonotic diseases in East Africa by country

More than half (52%) of the publications were on four (trypanosomiasis, brucellosis, Rift Valley Fever and rabies) of 22 diseases studied with Kenya, Tanzania, Uganda, Ethiopia, Rwanda and Burundi, contributing 39%, 22%, 21%, 15%, 2% and 1% of the total number of articles published respectively, Fig. 3. Publications on trypanosomiasis were mainly from Kenya and Uganda, Rift Valley fever mainly from Kenya with a few studies in Tanzania, Ebola primarily in Uganda, rabies in Tanzania and Ethiopia, patterns likely related to the incidence of the diseases in specific countries or interests in specific diseases by research groups working in those countries.

Overall, endemic zoonotic diseases accounted for more than 85% of all publications from the region, with publications on epidemic diseases such as Rift Valley fever, Ebola, Marburg and Middle East respiratory syndrome coronavirus associated with years when outbreaks of the diseases occurred in the region or elsewhere in the world (for example the Rift Valley fever epidemics of 2007/2008 in East Africa or MERS-CoV publications after the first reports of the disease in Saudi Arabia in 2012), Fig. 4.

**Fig. 4:**
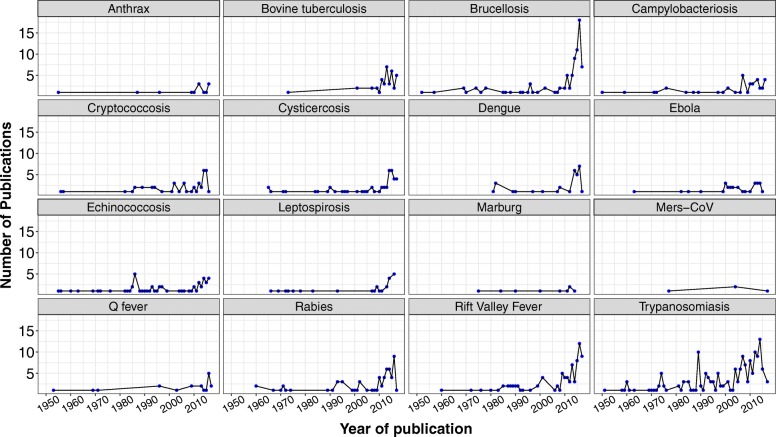
Diagram showing number of publications for specific zoonotic diseases over time

### Thesis data research trends

The University of Nairobi, the first university to be established in the country, had 101 (60%) of the theses followed by Kenyatta University 32 (19%), Jomo Kenyatta University of Agriculture and Technology 26 (15%), Moi University 6 (4%) and Egerton University 3 (2%).

**Fig. 5:**
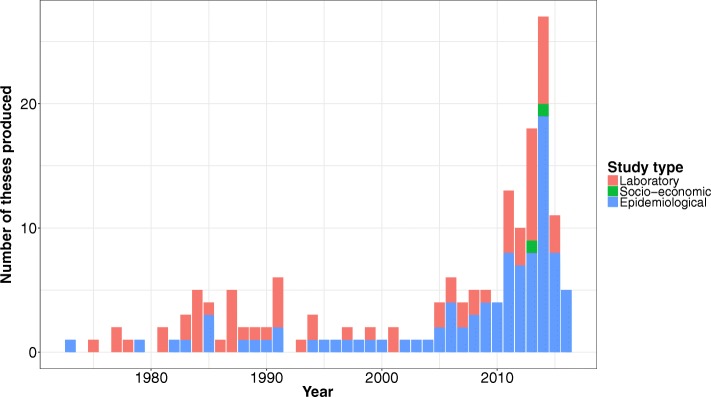
Diagram showing the number of theses on zoonotic diseases and the study type produced by the five main Kenyan Universities between 1970 and 2017

The number of theses produced per year increased with 102 (61%) of the theses produced in the last 10 years. Like the published data from the East Africa region, epidemiological studies were the most common accounting for 96 (57%), laboratory studies 70 (42%) and socio-economic 2 (1%) of all theses studies completed, Fig. 5.

Like the published data, majority 150 (89%) of theses on zoonoses in Universities in Kenya were on endemic diseases, with only a few focused on epidemic diseases Rift Valley fever 17 (10%) and Crimean-Congo hemorrhagic fever 1 (1%). By comparing the thesis research data and the published data for Kenya, we found only 11% of the theses research transitioned to peer-review publications.

**Fig. 6:**
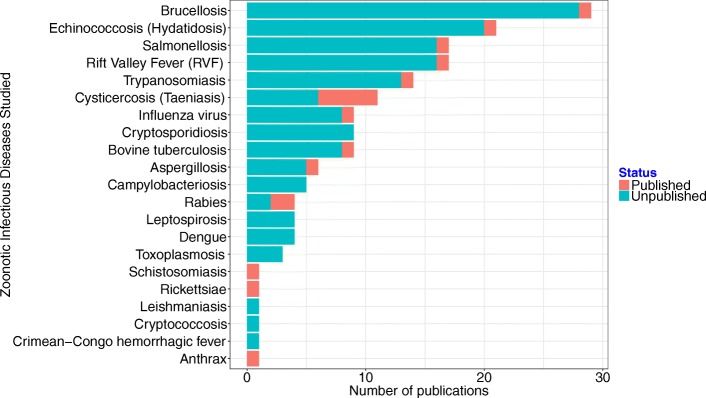
Figure showing the number of theses produced for each of the 21 zoonotic diseases studied in Kenya, and the number of theses research published in peer-reviewed journals

Among the 11% theses whose data was published in peer-reviewed journals, it took an average of 2.5 years from year the thesis was produced to publication of the manuscript (range 0–8). Figure 6 shows the total number of theses for each of the 21 zoonotic diseases evaluated and the proportion for each that were published in peer-reviewed scientific journals.

## Discussion

Our review of research trends on zoonotic diseases in East Africa has revealed a marked increase in publications and theses during the last decade, greater research focus on endemic diseases compared to epidemic diseases whose publications appear associated with specific disease outbreaks, focus on epidemiology type research and little of socio-economic studies, and a low and delayed transition of theses research into peer-reviewed journal publications.

For both journal publications from the East Africa region and theses production from Kenya on zoonotic diseases, two-thirds have come out in the last 10 years. This growth in zoonotic disease research is partly driven by the local and global recognition of the threats from zoonotic diseases and efforts to implement one health approaches in combating the public health and economic threats posed by these diseases. For example, Kenya established a One Health office referred to as the Zoonotic disease unit and one of its mandates is to stimulate and conduct research and training at the human-animal-ecosystem interfaces [15]. Most of the countries involved in this review including Kenya, Tanzania, Uganda, Rwanda and Ethiopia have established similar One Health offices or have organizations and networks created to conduct One Health training, research and outreach [16]. Publication output of countries has been associated with the level of national research spending and English proficiency [17]. These factors could be at play in East Africa region as well, with notable increase in the number of Universities established in the last 20 years [18]. The relatively fewer publications coming from Rwanda and Burundi may be associated with the two countries being French speaking, or political instability during the study period covered in our review.

Although much of the current global focus on zoonoses is mainly directed to emerging and re-emerging infectious diseases, our findings from both scientific and theses research highlight the importance of endemic zoonotic diseases compared to the epidemic prone emerging zoonoses such as Ebola, Marburg, highly pathogenic influenza virus and MERS-CoV in the region. This finding is supported by results of zoonotic disease prioritization carried out in Kenya and Ethiopia which have identified endemic zoonotic diseases as top priority [13, 19].

Publications on emerging zoonoses in the region were associated with outbreaks of the disease in the region or elsewhere in the world. Research on Rift Valley Fever heightened following the 2006/2007 outbreaks of the disease that occurred in Tanzania and Kenya [20]. The upward trend in publications on Ebola correspond to the Ebola outbreaks that occurred in various places in Uganda in 2000, 2007, 2011, and 2012 [21–24]. Such peaks in publications associated with disease outbreaks have been reported elsewhere including outbreaks of Severe Acute Respiratory syndrome (SARS) in Asia, Ebola in West Africa and MERS-CoV in the Middle East [25].

Although the bulk of the published research on zoonotic diseases is epidemiological, the finding that three-quarters of the studies were either case reports or cross-sectional studies limits utility of the data to mainly prevalence estimation and determination of associations between exposures and outcomes. Longitudinal studies, although more expensive and taking longer to conduct, allow for estimation of incidence rates, inference of causation and understanding transmission dynamics of infectious diseases [26]. Although both endemic and emerging zoonotic diseases have been associated with large public health and economic losses [3, 4, 27, 28], only a small proportion of the studies from the region have focused on socio-economics. Experiences from recent outbreak in zoonotic diseases in Africa revealed the need for studies that advance disease detection, diagnosis, predicting risk, understanding disease transmission dynamics, pathogen phylogenetics and phylogeography, and inform likely outcomes of available interventions including social and economic factors [20, 29–33].

Our study included review of theses research in Kenyan universities and revealed that up to 89% of the research failed to transition to peer-reviewed publications. Coupled with the 2.5 years average time delay between thesis production and manuscript publication for the 11% that were published, our results demonstrate that a considerable amount of zoonotic research work in the country and probably in the larger East Africa are not widely disseminated or immediately available to public health practitioners or policy makers. It is not clear why such a situation exists but it may be related to researchers’ only being keen to obtain an academic qualification or lack of proper academic support towards peer review publication. Recent change in policy on post-graduate education in Kenya making it mandatory for post-graduate students to show evidence of publication before being allowed to graduate may change the trend in dissemination of research findings, including those on zoonotic diseases.

## Conclusion

There is a marked increase in the number of research studies on zoonoses in the region mostly focused on endemic diseases, with publications on epidemic diseases triggered by outbreaks. Aspects of diseases such as incidence, economic impact, diagnostics and transmission dynamics are not given much attention and this remains a major knowledge gap. This improved research will lead to better public health interventions that reduce transmission and disease burden, and improve the well-being of both human and livestock populations in the region.

## Abbreviations

MERS-CoV: Middle East Respiratory Syndrome Coronavirus
RVF: Rift Valley fever
SARS: Severe Acute Respiratory syndrome
